# Supplementary material for: Antimicrobial Peptides SET-M33L and SET-M33L-PEG Are Promising Agents Against Strong Biofilm-Forming P. aeruginosa, Including Multidrug-Resistant Isolates
Source: Antibiotics (Basel). 2025 Jul 11;14(7):699. doi: 10.3390/antibiotics14070699 (PMC12291782; doi:10.3390/antibiotics14070699)
Supplement: Supplementary file 1 [file antibiotics-14-00699-s001.zip › antibiotics-3707226-supplementary.pdf]

Supplemental Data

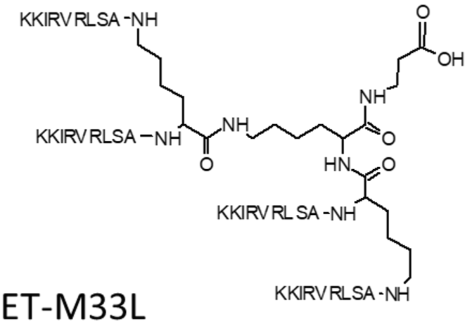

SET-M33L

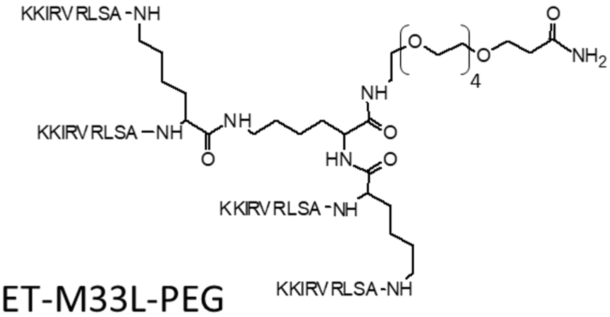

SET-M33L-PEG

Supplemental Figure S1. Chemical structure of SET-M33-L and SET-M33L-PEG.

Table S1. Minimum Inhibitory Concentration (MIC) and Minimum Bactericidal Concentration (MBC) of SET-M33 AMPs and conventional antimicrobials on 10 strong biofilm forming *P. aeruginosa* isolates.

Strain	Antimicrobial compounds μg/ml							
	**Tobramycin**		**Ceftazidime**		**SET-M33L**		**SET-M33L-PEG**	
	**MIC**	**MBC**	**MIC**	**MBC**	**MIC**	**MBC**	**MIC**	**MBC**
**PAO1**	2	4	2	2	16	64	16	64
**27547**	2	4	1	4	8	32	4	16
**31008**	2	8	2	8	64	128	32	128
**2002**	4	8	2	4	8	32	8	32
**72774**	4	8	2	4	8	32	8	32
**30375**	2	8	1	4	64	256	64	256
**30229**	2	8	4	16	32	128	8	32
27369	1	4	16	32	8	16	4	16
30475	2	8	32	128	2	8	2	4
27039	256	512	32	64	32	64	64	128
30429	16	32	8	64	16	64	32	64

MICs and MBCs values were obtained according to EUCAST guidelines with slight modifications, as described in Section 2.4. The four isolates labeled in italics represent MDR strains, resistant to three or more antimicrobial classes.

Table S2. Minimum and Maximum Minimum Biofilm Inhibitory Concentration (MBIC) of SET-M33 AMPs and conventional antibiotics on 10 strong biofilm forming *P. aeruginosa* isolates.

Strain	**Antimicrobial compounds μg/ml**							
	**Tobramycin**		**Polymyxin B**		**SET-M33L**		**SET-M33L-PEG**	
	**MBIC min**	**MBIC max**	**MBIC min**	**MBIC max**	**MBIC min**	**MBIC max**	**MBIC min**	**MBIC max**
**PAO1**	2	4	2	4	16	32	16	32
**27547**	1	2	0.5	1	8	16	4	8
**31008**	4	8	1	2	32	64	32	64
**2002**	8	16	1	2	32	64	32	64
**72774**	8	16	1	2	32	64	32	64
**30375**	4	8	-	>128	64	128	64	128
**30229**	2	4	0.5	1	32	64	16	32
27369	2	4	0.5	1	16	32	8	16
30475	8	16	0.25	0.5	4	8	2	4
27039	64	128	2	4	32	64	64	128
30429	32	64	1	2	64	128	32	64

MBICs values were obtained through crystal violet methodology, as described in Section 2.5. The four isolates labeled in italics represent MDR strains, resistant to three or more antimicrobial classes.

Table S3. Viability reduction in MBIC ranges of SET-M33 AMPs and conventional antimicrobials on 10 strong biofilm forming *P. aeruginosa* isolates

Strain	**Untreated**	**Tobramycin**				**Polymyxin B**				
**PAO1**	**0**	**1**	**2**	**4**	**8**	**1**	**2**	**4**	**8**	**μg/ml**
	1×10^{8}	1×10^{9}	1×10^{7}	1×10^{4}	0.0	1×10^{8}	1×10^{8}	1×10^{4}	1×10^{3}	**CFU/ml**
**27547**	**0**	**0.5**	**1**	**2**	**4**	**0.25**	**0.5**	**1**	**2**	**μg/ml**
	1×10^{7}	1×10^{7}	1×10^{6}	1×10^{5}	0.0	1×10^{7}	1×10^{6}	1×10^{3}	0.0	**CFU/ml**
**31008**	**0**	**2**	**4**	**8**	**16**	**0.5**	**1**	**2**	**4**	**μg/ml**
	1×10^{9}	1×10^{8}	1×10^{8}	1×10^{6}	1×10^{3}	1×10^{9}	1×10^{9}	1×10^{9}	1×10^{8}	**CFU/ml**
**2002**	**0**	**4**	**8**	**16**	**32**	**0.5**	**1**	**2**	**4**	**μg/ml**
	1×10^{8}	1×10^{7}	1×10^{6}	0.0	0.0	1×10^{8}	1×10^{7}	1×10^{4}	1×10^{3}	**CFU/ml**
**72774**	**0**	**4**	**8**	**16**	**32**	**0.5**	**1**	**2**	**4**	**μg/ml**
	1×10^{8}	1×10^{7}	1×10^{6}	0.0	0.0	1×10^{8}	1×10^{7}	1×10^{4}	1×10^{3}	**CFU/ml**
**30375**	**0**	**2**	**4**	**8**	**16**	**32**	**64**	**128**	**256**	**μg/ml**
	1×10^{8}	1×10^{8}	1×10^{8}	1×10^{8}	1×10^{8}	1×10^{8}	1×10^{8}	1×10^{8}	1×10^{8}	**CFU/ml**
**30229**	**0**	**1**	**2**	**4**	**8**	**0.5**	**1**	**2**	**4**	**μg/ml**
	1×10^{8}	1×10^{7}	1×10^{7}	1×10^{5}	1×10^{4}	1×10^{7}	1×10^{7}	1×10^{7}	1×10^{5}	**CFU/ml**
27369	**0**	**1**	**2**	**4**	**8**	**0.25**	**0.5**	**1**	**2**	**μg/ml**
	1×10^{7}	1×10^{7}	1×10^{7}	1×10^{5}	1×10^{2}	1×10^{6}	1×10^{6}	1×10^{6}	1×10^{3}	**CFU/ml**
30475	**0**	**4**	**8**	**16**	**32**	**0.25**	**0.5**	**1**	**2**	**μg/ml**
	1×10^{7}	1×10^{6}	0.0	0.0	0.0	1×10^{7}	1×10^{6}	1×10^{2}	0.0	**CFU/ml**
27039	**0**	**32**	**64**	**128**	**256**	**1**	**2**	**4**	**8**	**μg/ml**
	1×10^{6}	1×10^{7}	1×10^{6}	1×10^{4}	0.0	1×10^{6}	1×10^{5}	1×10^{2}	0.0	**CFU/ml**
30429	**0**	**16**	**32**	**64**	**128**	**0.5**	**1**	**2**	**4**	**μg/ml**
	1×10^{8}	1×10^{7}	1×10^{5}	0.0	0.0	1×10^{8}	1×10^{7}	1×10^{7}	1×10^{5}	**CFU/ml**

Strain	**Untreated**	**SET-M33L**				**SET-M33L-PEG**				
**PAO1**	**0**	**8**	**16**	**32**	**64**	**8**	**16**	**32**	**64**	**μg/ml**
	1×10^{8}	1×10^{8}	1×10^{8}	1×10^{5}	0.0	1×10^{8}	1×10^{8}	1×10^{5}	0.0	**CFU/ml**
**27547**	**0**	**4**	**8**	**16**	**32**	**2**	**4**	**8**	**16**	**μg/ml**
	1×10^{7}	1×10^{8}	1×10^{6}	1×10^{4}	1×10^{3}	1×10^{7}	1×10^{7}	1×10^{4}	1×10^{3}	**CFU/ml**
**31008**	**0**	**16**	**32**	**64**	**128**	**16**	**32**	**64**	**128**	**μg/ml**
	1×10^{9}	1×10^{8}	1×10^{8}	1×10^{6}	1×10^{3}	1×10^{8}	1×10^{8}	1×10^{5}	1×10^{3}	**CFU/ml**
**2002**	**0**	**16**	**32**	**64**	**128**	**16**	**32**	**64**	**128**	**μg/ml**
	1×10^{8}	1×10^{8}	1×10^{8}	1×10^{5}	0.0	1×10^{9}	1×10^{8}	1×10^{4}	0.0	**CFU/ml**
**72774**	**0**	**16**	**32**	**64**	**128**	**16**	**32**	**64**	**128**	**μg/ml**
	1×10^{8}	1×10^{8}	1×10^{8}	1×10^{5}	0.0	1×10^{9}	1×10^{8}	1×10^{4}	0.0	**CFU/ml**
**30375**	**0**	**32**	**64**	**128**	**256**	**32**	**64**	**128**	**256**	**μg/ml**
	1×10^{7}	1×10^{7}	1×10^{6}	1×10^{3}	0.0	1×10^{7}	1×10^{7}	1×10^{5}	0.0	**CFU/ml**
**30229**	**0**	**16**	**32**	**64**	**128**	**8**	**16**	**32**	**64**	**μg/ml**
	1×10^{8}	1×10^{8}	1×10^{8}	1×10^{7}	1×10^{4}	1×10^{8}	1×10^{8}	1×10^{8}	1×10^{5}	**CFU/ml**
27369	**0**	**8**	**16**	**32**	**64**	**4**	**8**	**16**	**32**	**μg/ml**
	1×10^{7}	1×10^{8}	1×10^{8}	1×10^{7}	1×10^{2}	1×10^{7}	1×10^{7}	1×10^{4}	1×10^{3}	**CFU/ml**
30475	**0**	**2**	**4**	**8**	**16**	**1**	**2**	**4**	**8**	**μg/ml**
	1×10^{7}	1×10^{7}	1×10^{5}	1×10^{3}	1×10^{2}	1×10^{7}	1×10^{6}	1×10^{5}	1×10^{3}	**CFU/ml**
27039	**0**	**16**	**32**	**64**	**128**	**32**	**64**	**128**	**256**	**μg/ml**
	1×10^{6}	1×10^{6}	1×10^{6}	1×10^{6}	1×10^{3}	1×10^{6}	1×10^{6}	1×10^{4}	0.0	**CFU/ml**
30429	**0**	**32**	**64**	**128**	**256**	**16**	**32**	**64**	**128**	**μg/ml**
	1×10^{8}	1×10^{8}	1×10^{5}	1×10^{3}	1×10^{3}	1×10^{8}	1×10^{7}	1×10^{2}	0.0	**CFU/ml**

Viability was measured through colony counting by plating of a pre-treated biofilm with AMP or conventional antimicrobial concentrations ranging from 0.5 x MBIC to 2 x MBIC for each of the respective treatments. The four isolates labeled in italics represent MDR strains, resistant to three or more antimicrobial classes.

Table S4. Clinical metadata of clinical *P. aeruginosa* isolates.

Isolate	Date of Isolation	Place of isolation	Year of isolation	Pulmonary-related disease
**27547**	26-Jul	Anonymous	2023	CF
**A13L9**	Unknown	Anonymous	2013	Unknown
**60002**	03-Aug	Anonymous	2023	CF
**31008**	12-Jul	Anonymous	2024	CF
**22102**	27-Jun	Anonymous	2024	CF
**66702**	09-Aug	Anonymous	2023	CF
**2002**	26-Jun	Anonymous	2024	CF
**72774**	26-Jun	Anonymous	2024	CF
**30814**	19-Jun	Anonymous	2024	CF
**30375**	30-Apr	Anonymous	2024	CF
**30229**	21-Apr	Anonymous	2024	CF
**27369**	12-Jul	Anonymous	2023	Bronchiectasis
**30475**	08-May	Anonymous	2024	CF
**27039**	29-May	Anonymous	2023	CF
**30806**	16-Jun	Anonymous	2024	CF
**30429**	10-May	Anonymous	2024	CF
**72387**	08-May	Anonymous	2024	CF

CF=Cystic Fibrosis

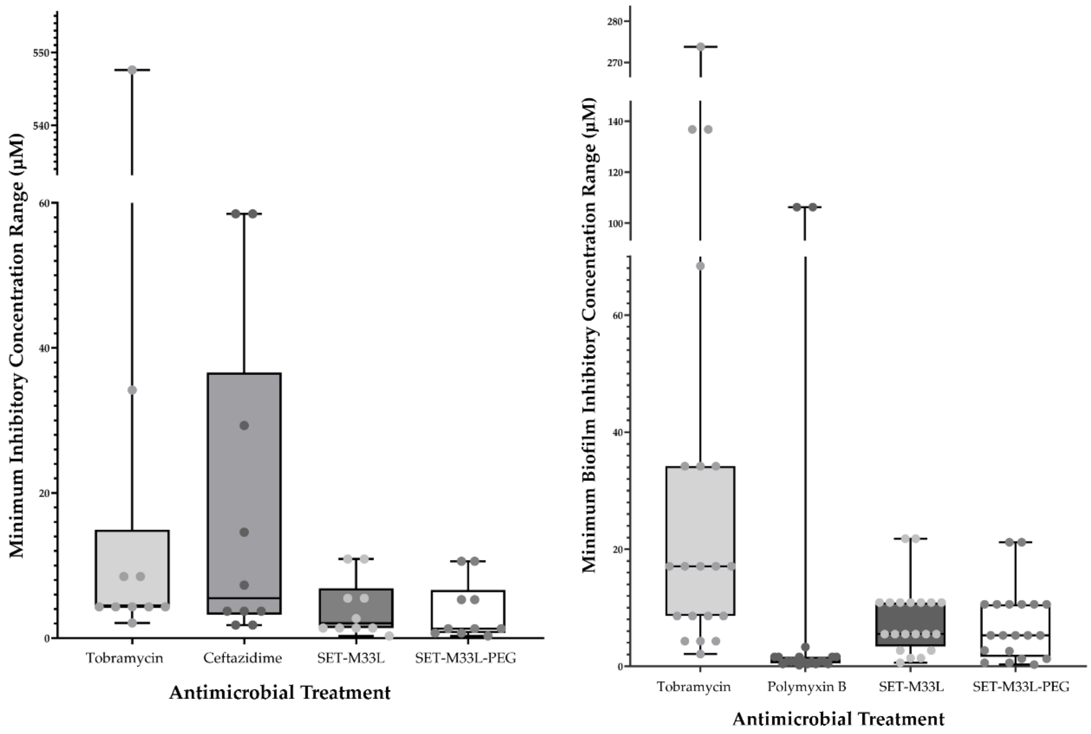

Supplemental Figure S2. Minimum Inhibitory Concentrations and Minimum Bactericidal Concentrations of SET-M33 AMPs against ten clinical isolates of *P. aeruginosa* in comparison with conventional antibiotics tobramycin and ceftazidime. Data are displayed as ranges with median values among the ten isolates**.**

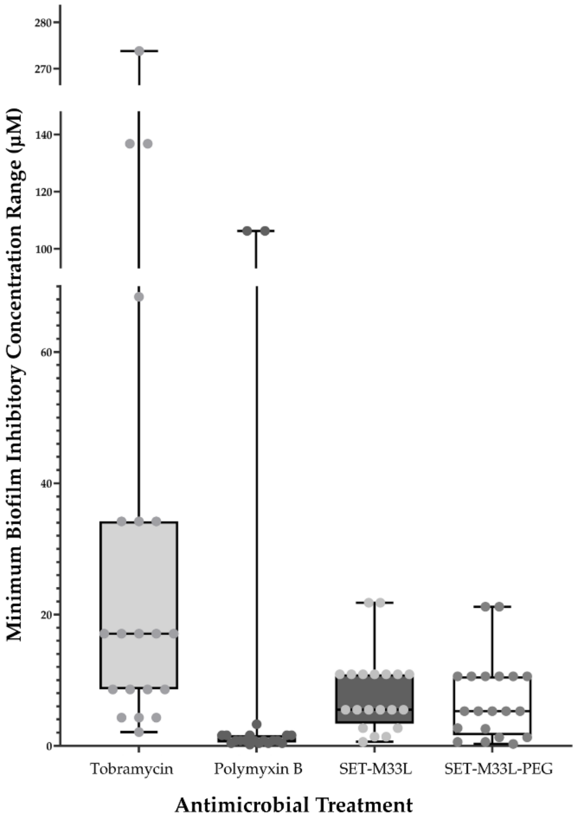

Supplemental Figure S3. Minimum Biofilm Inhibitory Concentrations of SET-M33 AMPs against ten clinical isolates of *P. aeruginosa* in comparison with conventional antibiotic tobramycin and AMP already in clinical use Polymyxin B. Data are displayed as ranges with median values among the ten isolates.
